# Racial and Socioeconomic Disparities in Out-Of-Hospital Cardiac Arrest Outcomes: Artificial Intelligence-Augmented Propensity Score and Geospatial Cohort Analysis of 3,952 Patients

**DOI:** 10.1155/2021/3180987

**Published:** 2021-11-24

**Authors:** Dominique J. Monlezun, Alfred T. Samura, Ritesh S. Patel, Tariq E. Thannoun, Prakash Balan

**Affiliations:** ^1^Department of Cardiology, The University of Texas MD Anderson Cancer Center, Houston, TX, USA; ^2^Center for Artificial Intelligence and Health Equities, Global System Analytics & Structures, Bethesda, MD, USA; ^3^Department of Cardiology, Cooper Medical School of Rowan University, Camden, NJ, USA; ^4^Division of Cardiovascular Sciences, University of South Florida Morsani College of Medicine, Tampa, FL, USA; ^5^Section of Cardiology, Tulane University School of Medicine, New Orleans, LA, USA; ^6^University of Arizona College of Medicine at Phoenix, Department of Cardiology, Phoenix, AZ, USA

## Abstract

**Introduction:**

Social disparities in out-of-hospital cardiac arrest (OHCA) outcomes are preventable, costly, and unjust. We sought to perform the first large artificial intelligence- (AI-) guided statistical and geographic information system (GIS) analysis of a multiyear and multisite cohort for OHCA outcomes (incidence and poor neurological disposition).

**Method:**

We conducted a retrospective cohort analysis of a prospectively collected multicenter dataset of adult patients who sequentially presented to Houston metro area hospitals from 01/01/07-01/01/16. Then AI-based machine learning (backward propagation neural network) augmented multivariable regression and GIS heat mapping were performed.

**Results:**

Of 3,952 OHCA patients across 38 hospitals, African Americans were the most likely to suffer OHCA despite representing a significantly lower percentage of the population (42.6 versus 22.8%; *p* < 0.001). Compared to Caucasians, they were significantly more likely to have poor neurological disposition (OR 2.21, 95%CI 1.25–3.92; *p*=0.006) and be discharged to a facility instead of home (OR 1.39, 95%CI 1.05–1.85; *p*=0.023). Compared to the safety net hospital system primarily serving poorer African Americans, the university hospital serving primarily higher income commercially and Medicare insured patients had the lowest odds of death (OR 0.45, *p* < 0.001). Each additional $10,000 above median household income was associated with a decrease in the total number of cardiac arrests per zip code by 2.86 (95%CI -4.26- -1.46; *p* < 0.001); zip codes with a median income above $54,600 versus the federal poverty level had 14.62 fewer arrests (*p* < 0.001). GIS maps showed convergence of the greater density of poor neurologic outcome cases and greater density of poorer African American residences.

**Conclusion:**

This large, longitudinal AI-guided analysis statistically and geographically identifies racial and socioeconomic disparities in OHCA outcomes in a way that may allow targeted medical and public health coordinated efforts to improve clinical, cost, and social equity outcomes.

## 1. Introduction

Out-of-hospital cardiac arrest (OHCA) remains a serious medical and public health challenge in the United States. Approximately 350,000 OHCAs occur annually with survival rates of less than 10% [1,2]. Improving outcomes requires building a chain of prevention through improved comorbidity management and survival that begins with immediate bystander cardiopulmonary resuscitation (CPR), early and rapid deployment of emergency medical services (EMS), the use of automated external defibrillators (AEDs), and prompt triage to a resuscitation capable hospital with hemodynamic support and percutaneous coronary intervention capabilities, as well as the necessary critical care infrastructure to provide postarrest care [3–10]. Despite efforts to standardize optimal cardiac arrest care, numerous disparities persist by race and socioeconomics, thus highlighting the need for coordinated medical and public health efforts to improve clinical, cost, and social equity outcomes [11–21].

Such previous studies have provided important clinical insights to clarify how racial and socioeconomic factors may be significant and independent predictors of disparities in OHCA outcomes (even controlling for known demographic and comorbidities predicting worse outcomes). Separately, a recent geospatial or geographic information system (GIS) analysis provided a seminal representative example of how OHCA incidence can be mapped relative to race and income yet without robust attempts to reduce confounders [22]. Yet, such disparity results are difficult to translate into effective and comprehensive countermeasures, as evidenced by the largely unchanged poor OHCA outcomes and persistent social inequities. To achieve appropriate countermeasures, an approach integrating both statistical and geographic analyses is required to clarify the causal pipeline of outcomes, where prevention should focus, where EMS can improve transportation times, which hospitals can better coordinate with that mentioned above and implement improved OHCA standardized care protocols and how the above health system can better transition survivors back into their communities safely for optimal changes of improvement. Yet, given the complexity of such data (in type, source, and rapid timing) and the growing success of newer artificial intelligence- (AI-) guided methodologies to answer such challenges, we sought to perform the first large and integrated AI-guided causal inference statistical and geographic information system (GIS) analysis of a multiyear and multisite cohort for OHCA outcomes (in incidence, mortality, and poor neurological disposition).

## 2. Methods

This multisite prospective cohort examined cardiac arrest subjects across the Houston metro from 01/01/07–01/01/16. Multivariable regression was then conducted according to the method of machine learning-augmented modified propensity score-adjusted multivariable regression (ML-PSr) [23–25] in three phases. First, overall sample descriptive statistics and bivariable analysis by inpatient mortality were conducted: continuous variables were analyzed by means using an independent-sample t-test and by medians using Wilcoxon rank sum tests, and categorical variables were analyzed by proportions using Pearson's chi square test or Fisher's exact test. Variables flagged for their statistical or clinical significance in the first step were then included in the tentative final models. Next, the variables were considered according to stepwise forward and backward regression. Last, performance for the final models before approving them was considered according to regression diagnostics. These tests included machine learning performance comparison to the regression models' root mean squared error (RMSE), Akaike's and Schwarz's Bayesian information criteria, AUC, Hosmer–Lemeshow goodness-of-fit test, specification error, variance inflation factor, correlation matrix, multicollinearity, and tolerance.

ML-PSr was chosen as the primary computational methodology for the following reasons [23–25]. Its most basic framework is the well-accepted traditional statistical method of multivariable regression that allows significant and independent associations between predictors and outcomes to be assessed in a way that is familiar to most biomedical researchers. Next, it integrates the well-accepted traditional statistical and causal inference technique of propensity score adjustment to postulate not just association but potential causality in nonrandomized data by reducing such biases as confounding and selection biases which threaten study validity (while noting that randomized controlled trials are the gold standard for assessing real causality, but successful ones historically are launched only after sufficient nonrandomized and particularly causal inference studies better clarify the hypothesis to be tested). The rationale for using adjustment in contrast to other propensity score methods is detailed in the abovementioned references. Finally, the use of machine learning as a hybrid approach integrated with the abovementioned traditional statistical results has already been extensively documented, including its benefit of reproducing the abovementioned results while going beyond that to incorporate more rapid and even real-time analysis using larger and higher dimension data from more varied sources. The net outcome from that mentioned above is allowing a bridge from traditional statistics to the increasingly well-accepted supervised machine learning with implications for unsupervised and general artificial intelligence to allow more rapid and still reliable results to guide more effective diagnosis and treatment of patients.

An academic biostatistician and physician-data scientist (DJM) reviewed the final models to confirm they were sufficiently supported by clinical and statistical theory and the current literature prior to approving them as the final models, which were, thus, reported as fully adjusted results with 95% confidence intervals (CIs). The final regression models fully adjusted for prehospital outcomes (including age, arrest traits (asystole, location, occurrence before first responder arrival, and unwitnessed), presenting hospital, and the likelihood of presenting at the safety net hospital) to better allow racial disparity assessment, particularly in relation to timely healthcare access. Statistical significance was set with a two-tailed *p* value <0.05. STATA 14.2 (STATACorp, College Station, Texas, USA) performed statistical analyses, and Java 9 (Oracle, Redwood Chores, California, USA) ran machine-learning algorithms. Geospatial analysis was conducted with neural network multivariable regression of arrest outcomes.

## 3. Results

Of 3,952 OHCA patients across 38 hospitals, the mean (standard deviation) age in years was 63.21 (15.44), 1,654 (41.85%) were female, 2,619 (66.27%) were nonwhite, 2,639 (66.78%) ultimately died by hospital discharge, and 296 (72.20%) had poor neurological outcome (Table 1). Nonwhite patients with OHCA were significantly more likely to be younger (mean [SD] 62.03 [15.17] versus 65.53 [15.70]) and female (45.06% versus 35.56%), in addition to being significantly less likely to have bystander CPR (27.35% versus 36.59%), no AED (67.63% versus 58.56%), and asystole as the presenting rhythm (68.27% versus 59.19%).

African Americans specifically were the most likely to suffer OHCA despite representing a significantly lower percentage of the population (42.6 versus 22.8%; *p* < 0.001).

By zip code, there were 16.92 (SD 21.55) arrests on average. Regression analysis showed each additional $10,000 above median household income was associated with a decrease in the total number of cardiac arrests per zip code by 2.86 (95%CI −4.26–1.46; *p* < 0.001); zip codes with a median income above $54,600 versus the federal poverty level lowered arrests by 14.62 (*p* < 0.001).

**Figure 1:**
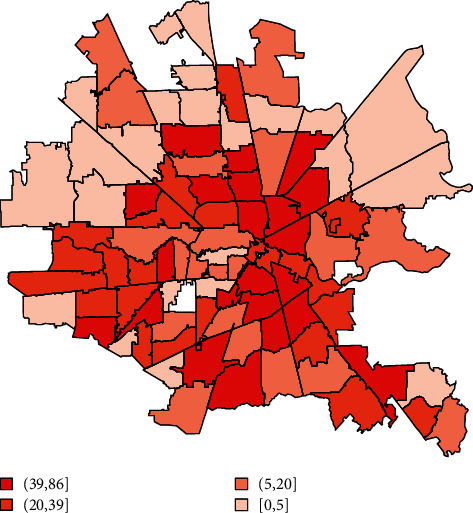
Cardiac arrest mortality by the Houston metro zip code (*N* = 3,952).

**Figure 2:**
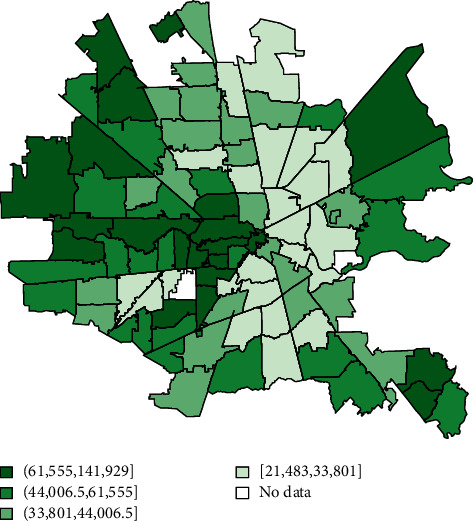
Median income by the Houston metro zip code (*N* = 3,952).

**Figure 3:**
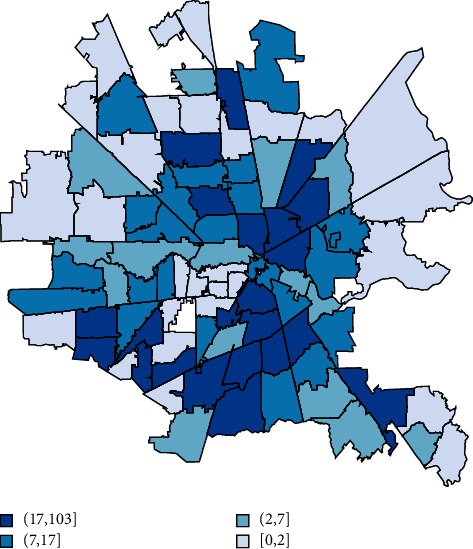
African Americans with cardiac arrest by the Houston metro zip code (*N* = 3,952).

**Figure 4:**
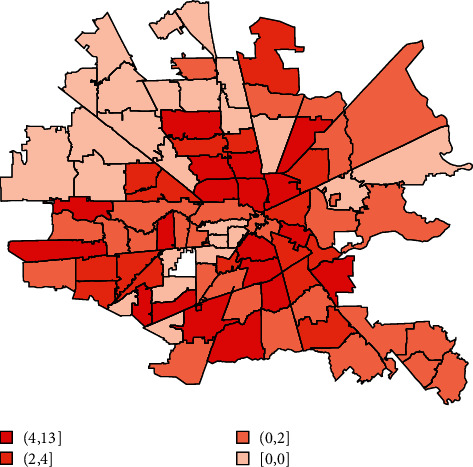
Poor neurological outcome after cardiac arrest by the Houston metro zip code (*N* = 3,952).

**Figure 5:**
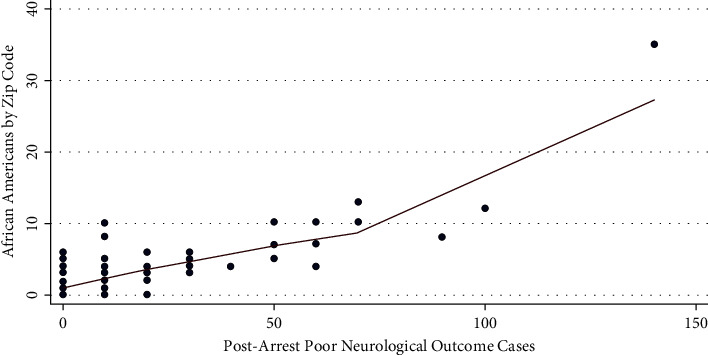
Association of African Americans versus poor neurologic outcomes after cardiac arrest by locally weighted regression (LOWESS) (*N* = 3,952).

For each additional 10 African Americans suffering cardiac arrest in a zip code, its total number of poor neurologic outcomes increased by an average of 8.78 (*p* < 0.001). Geospatial maps showed a clockwise band from north to east to south of higher cardiac arrest, associated mortality, lower income, and more poor neurologic outcome cases overlapping with where more African Americans with lower incomes lived (Figures 1–4). The association of race and poor neurological outcome was additionally confirmed by locally weighted regression (Figure 5).

**Figure 6:**
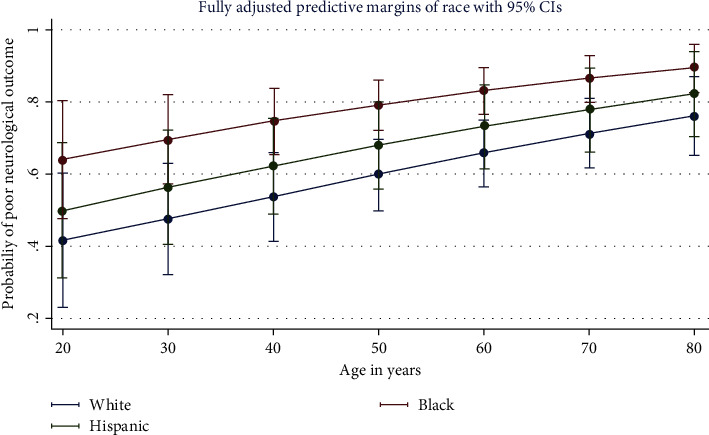
Post-cardiac arrest neurologic outcomes by race in multivariable regression (*N* = 3,952).

The top independent predictors of inpatient mortality after cardiac arrest were asystole (OR 3.81, 95%CI 3.26–4.44; *p* < 0.001), cardiac arrest before 911 was dialed (OR 1.90, 95%CI 1.56–2.30; *p* < 0.001), and an unwitnessed arrest (OR 1.80, 95%CI 1.51–2.15; *p* < 0.001). Significant racial disparities were detected. African Americans compared to Caucasians were significantly less likely to die inpatient (OR 0.84, 95%CI 0.71–1.00; *p*=0.046), but were significantly more likely to have poor neurological outcome (OR 2.21, 95%CI 1.25–3.92; *p*=0.006) (Figure 6) and be discharged to long-term acute care facilities (LTACs) or skilled nursing facilities (SNFs) instead of home (OR 1.39, 95%CI 1.05–1.85; *p*=0.023). Hispanics had similar outcomes to Caucasians.

There were no significant racial disparities in time from cardiac arrest to EMS arrival. Compared to the safety net hospital system, the university hospital serving largely commercially and Medicare-insured patients had the lowest odds of death (OR 0.45, *p* < 0.001) followed by the main private hospital primarily serving commercially insured patients (OR 0.62, *p*=0.017). Propensity score-adjusted multivariable regression produced similar results.

## 4. Discussion

Our study is the first known large, longitudinal, multicenter, and integrated AI-guided causal inference statistical and geospatial analysis of racial disparities in OHCA incidence and outcomes. This study provides not only novel evidence of the overlapping geographic density of poorer African Americans with higher incidence of OHCA and worse neurological outcomes (with greater association of income rather than race) but also computational evidence that causal inference may apply to this association. It further suggests that regardless of clinical risk factors and EMS transport times, hospitals may have superior OHCA outcomes if they primarily serve higher income and better insured patients. These results may inform concrete countermeasures including targeted and culturally sensitive health system interventions to improve health outcomes and inequities by better understanding, for instance, how community clinics, schools, churches, and other cultural organizations may be integrated with larger health system efforts to provide community-preferred longitudinal access to high-quality and affordable care, health education, and early identification of patients at higher risk of cardiac arrest requiring more aggressive prevention and postarrest care.

Our study provides additional comprehensive and complementary insights compared to earlier studies [22,26–28] including the landmark ARIC study [26]. In it, the lifetime cumulative incidence of sudden cardiac death (SCD) at age 85 years was 1.5 times greater in black men compared to white men and almost 3 times greater in black women compared to white women. Region-specific data from large metros additionally support such disparities. Galea et al. examined 4,000 cases of out-of-hospital SCD with EMS response and attempted resuscitation in New York City, demonstrating that the age-adjusted SCD rate among African Americans was 40% higher than among Caucasians [27]. In Seattle, Cowie et al. found African Americans were two times more likely to suffer from nontraumatic SCD than whites [28]. In Chicago, the SCD rate is up to twice as high [29]. Similar results were obtained in San Francisco and Oregon [30,31]. Part of these disparities may be attributed to differences in EMS response times based on socioeconomic status [14]. However, in our study, we found no significant racial disparities in response times.

Socioeconomic factors increasingly appear to play a predominant role in OHCA disparities (and, thus, certain races have higher incidences of lower median incomes and, thus, may have correlative worse outcomes). Similar to the work of Raun et al. [22], we found lower-income areas in Houston (households making less than the median household income of $55,000) also happened to be predominantly African American and have a higher incidence of cardiac arrest compared to areas with higher annual household incomes. Our study also corroborates the findings of the ARIC study which suggests that income inequality is a significant contributor of disparities in OHCA incidence [26]. Additional studies are underway to clarify how socioeconomic status and race may interact in a complex fashion to drive the abovementioned outcomes, such as certain races being more likely to have lower rates of adequate healthcare insurance, health system access, and comorbidity management, therefore leading to cardiac arrest as the first clear manifestation of insufficiently managed chronic conditions.

Unlike prior studies, however, our study provides a more granular geographic and statistical understanding of the racial and socioeconomic disparities in OHCA outcomes in a typical urban American population. Our analysis revealed novel insights into the differences in incidence and outcome (with sufficient detail to differentiate between mortality and poor neurological outcomes) including differences by the type of treatment facility to which cardiac arrests were triaged.

Surprisingly in our study, African Americans compared to Caucasians were significantly less likely to die inpatient (OR 0.84, 95%CI 0.71–1.00; *p*=0.046) after suffering cardiac arrest. Yet, our analysis also showed poorer neurologic outcomes among blacks as compared to whites (OR 2.21, 95%CI 1.25–3.92; *p*=0.006). Furthermore, among those surviving to discharge, blacks were more likely to be discharged to LTAC or SNF rather than home as compared to whites. This phenomenon has not previously been well described. We suspect that this finding demonstrates at least in part subtle underlying cultural differences in goals of care. Prior research has suggested that that African American more often favor continuing life-prolonging measures as compared to Caucasians regardless of the projected quality of life [32,33]. Cultural differences in attitudes toward end-of-life care have been described in a number of contexts [32,33] but have not been sufficiently explored in cardiac arrest care. Our findings shed light on these potential subtle cultural differences and underscore the importance of medical teams respecting such important cultural differences.

Our analysis also provides insights into OHCA outcomes based on the type of hospital rendering cardiac arrest care. Previous studies have noted divergent outcomes based on the racial composition of hospitalized populations [34,35]. We found significant differences in overall death among different hospital systems even controlling for severity of illness and prehospitalization OHCA traits and EMS care. Compared with the safety net county hospital system (principally serving lower income and underinsured patients), the university hospital principally serving higher income commercially and Medicare insured patients had the lowest odds of death followed by the main private hospital primarily serving commercially insured patients. This may be related to differences in patient profiles for each system with county hospitals managing patients with less access to preventative healthcare as compared to the teaching and private hospitals serving patient populations with more access to longitudinal, preventative care. However, the tendency toward better outcomes in hospitals with more experience in cardiac arrest care has also been described [35]. Our findings suggest that the quaternary-care hospitals with greater resources and experience in cardiac arrest care may be best equipped to manage cardiac arrest patients. In light of the racial and socioeconomic disparities in cardiac arrest outcomes, replication and sharing of treatment processes used at quaternary-care facilities at other medical centers may provide some means of mitigating disparities in outcomes.

The strengths of our analysis include its large, multicenter, longitudinal cohort, as well as more comprehensive geospatial and causal inference statistics augmented by machine learning, which allows more sophisticated analysis of more complex data closer to real time. Such strengths were meant to reduce the associated study limitations and external validity threats including bias due to nonrandomized data focused on a single metro area with lack of more granular data regarding chronic comorbidity and self-identifying culturally conditioned preferences in care.

## 5. Conclusions

This is the first known large, longitudinal, multicenter, and integrated AI-guided causal inference statistical and geospatial analysis of racial disparities in OHCA outcomes. This study provides novel evidence of the independent and potentially even causal relationship between racial socioeconomics and OHCA outcomes in a way that may allow targeted countermeasures to improve health and equity outcomes at the preventive and therapeutic levels.

## Figures and Tables

**Table 1:** Descriptive and bivariable analysis by the nonwhite race in cardiac arrest (*N* = 3,952)^*∗*^.

Variable no. (%)	Sample, *N* = 3,952	White, *N* = 1,333 (33.73%)	Nonwhite, *N* = 2,619 (66.27%)	*p* value
*Demographics*				
Age, mean (SD)	63.21 (15.44)	65.53 (15.70)	62.03 (15.17)	<0.001
Female	1,654 (41.85)	474 (35.56)	1,180 (45.06)	<0.001

Race				<0.001
White	1,333 (33.73)	1,333 (100.00)	0 (0.00)	
Black	1,666 (42.16)	0 (0.00)	1,666 (63.61)	
Hispanic	767 (19.41)	0 (0.00)	767 (29.29)	
Other	186 (4.71)	0 (0.00)	186 (7.10)	

*Arrest*				
No witness	1,291 (32.67)	424 (31.81)	867 (33.10)	0.411
Before 911	3,211 (81.25)	1,133 (85.00)	2,078 (79.34)	<0.001
Bystander CPR	1,203 (30.46)	487 (36.59)	716 (27.35)	<0.001
CPR delay	712 (51.82)	321 (61.97)	391 (45.68)	<0.001
No AED	1,049 (64.32)	349 (58.56)	700 (67.63)	<0.001
Asystole	2,577 (65.21)	789 (59.19)	1,788 (68.27)	<0.001

*Hospital*				
THP	1,118 (45.73)	363 (46.07)	755 (45.56)	0.816

Discharge				
Death	2,639 (66.78)	888 (66.62)	1,751 (66.86)	0.879
Nonhome	635 (48.77)	209 (47.29)	426 (49.53)	0.442
Poor neurological	296 (72.20)	87 (60.84)	209 (78.28)	<0.001

^*∗*^No., number; SD, standard deviation; CPR, cardiopulmonary resuscitation; AED, automated external defibrillator; ROSC, return of spontaneous circulation; THP, therapeutic hypothermic protocol.

## Data Availability

The Excel data used to support the findings of this study are available from the corresponding author upon request.
